# Secondary Data Analysis of National Surveys in Japan Toward Improving Population Health

**DOI:** 10.2188/jea.JE20150319

**Published:** 2016-03-05

**Authors:** Nayu Ikeda

**Affiliations:** Center for International Collaboration and Partnership, National Institute of Health and Nutrition, National Institutes of Biomedical Innovation, Health and Nutrition, Tokyo, Japan

**Keywords:** secondary data analysis, national health survey, population health, Japan

## Abstract

Secondary data analysis of national health surveys of the general population is a standard methodology for health metrics and evaluation; it is used to monitor trends in population health over time and benchmark the performance of health systems. In Japan, the government has established electronic databases of individual records from national surveys of the population’s health. However, the number of publications based on these datasets is small considering the scale and coverage of the surveys. There appear to be two major obstacles to the secondary use of Japanese national health survey data: strict data access control under the Statistics Act and an inadequate interdisciplinary research environment for resolving methodological difficulties encountered when dealing with secondary data. The usefulness of secondary analysis of survey data is evident with examples from the author’s previous studies based on vital records and the National Health and Nutrition Surveys, which showed that (i) tobacco smoking and high blood pressure are the major risk factors for adult mortality from non-communicable diseases in Japan; (ii) the decrease in mean blood pressure in Japan from the late 1980s to the early 2000s was partly attributable to the increased use of antihypertensive medication and reduced dietary salt intake; and (iii) progress in treatment coverage and control of high blood pressure is slower in Japan than in the United States and Britain. National health surveys in Japan are an invaluable asset, and findings from secondary analyses of these surveys would provide important suggestions for improving health in people around the world.

## POPULATION HEALTH AND SECONDARY SURVEY DATA ANALYSIS

Population health is the aggregate of health conditions of individuals who share a natural, cultural, and social environment within a health system. In addition to clinical determinants, population health is subject to complex interactions of multiple internal and external forces, which are both human and uncontrollable. The scientific field of health metrics and evaluation reflects this characteristic of population health. Health metrics and evaluation pursues the assessment of health issues at the system level and integrates interdisciplinary knowledge from medicine, epidemiology, demography, economics, mathematics, and other relevant fields.^1^

Researchers in health metrics and evaluation typically analyze individual-level data from existing national household surveys to generate comparable evidence across countries and over time for monitoring trends in the health status of populations and benchmarking the performance of health systems. Use of survey data collected by others in the past for different purposes is preferable when it is financially or technically impossible for researchers to independently collect primary data from a sufficiently large sample for investigation of population health issues. Secondary analysis of national survey data dates back at least to the 1950s, and it originally occupied a central position in social science.^2^^,^^3^ In particular, national household surveys focus on households and residents in areas separated by political boundaries; such surveys collect information about residents’ lives in a social context, such as with respect to families, neighborhoods, schools, and workplaces. These characteristics of national household surveys are also useful in epidemiological studies exploring social aspects of health, including socioeconomic inequalities and social determinants. Moreover, national household surveys typically use complex survey designs, such as stratification, clustering, and sampling weights, to obtain a large, nationally representative sample of a target population group. It is appropriate to employ data of such probabilistic samples covering a countrywide population when assessing health problems of interest at the national and subnational levels.

Today, online access to freely available electronic records has facilitated the public use of individual-level data of national health surveys from such countries as the United Kingdom,^4^ the United States,^5^ and developing nations that participate in global initiatives, including the Demographic Health Surveys Program.^6^ A notable example of secondary survey data analysis is collaborative research on long-term trends in the global burden of metabolic risk factors for non-communicable diseases. Such studies have utilized existing individual-level data from national household surveys as a key data source,^7^ particularly with respect to measurements of body height and weight,^8^^,^^9^ systolic blood pressure,^10^ blood cholesterol,^11^ and blood glucose or hemoglobin A1c.^12^^–^^14^

In this Young Investigator Award Winner’s special article, I describe the current situation of population health research based on secondary data analysis of national surveys on the general population in Japan. I then present a few examples of secondary survey data analysis from my published studies to illustrate its usefulness in epidemiology in Japan. This article not only promotes understanding of readers overseas about institutional and technical challenges for scientific use of Japanese survey data but also encourages epidemiologists in Japan to advance knowledge on population health through exploring national survey data.

## SECONDARY DATA ANALYSIS OF NATIONAL HEALTH SURVEYS IN JAPAN

In Japan, the Ministry of Health, Labour and Welfare has developed electronic databases of individual records from the complete vital registration, as well as population and health surveys conducted on nationally representative samples of the general population (Table). Currently, the oldest electronically available data in Japan are death and stillbirth records for 1972 (Statistics and Information Department, Ministry of Health, Labour and Welfare, personal communication). These surveys enable researchers to explore a wide range of health indicators in depth, including fertility, mortality, morbidity, health service utilization, and health risks and behaviors, in Japan.

**Table.** Availability of secondary data of individual records from national surveys on population and health conducted on general population by the Ministry of Health, Labour and Welfare

Survey title	Secondary data availability^a^
Vital Statistics	Annual; Live birth records (1974–), Stillbirth records (1972–), Death records (1972–), Marriage records (1973–), Divorce records (1973–)^b^
National Survey on Migration	Every 5 years, 1991–^107^
National Fertility Survey	Every 5 years, 1977–2002, 2005–^107^
National Survey on Family	Every 5 years, 1993–^107^
National Survey on Household Changes	Every 5 years, 1994–^107^
Comprehensive Survey of Living Conditions	Household and income questionnaires (annual, 1986–), Health and savings questionnaires (every 3 years, 1986–), Long-term care questionnaire (every 3 years, 2001–)
Longitudinal Survey of Newborns in the 21st Century	Annual; 2001 Cohort (2001–), 2010 Cohort (2010–)
Longitudinal Survey of Adults in the 21st Century	Annual; 2002 Cohort (2002–), 2012 Cohort (2012–)
Longitudinal Survey of Middle-aged and Elderly Persons	Annual, 2005–
National Health and Nutrition Survey^c^	Annual, 1973–^d^

^a^As of October 20, 2015.

^b^Statistics and Information Department, Ministry of Health, Labour and Welfare, personal communication.

^c^Name changed from National Nutrition Survey in 2003.

^d^Health Service Bureau, Ministry of Health, Labour and Welfare, personal communication.

An increasing number of original research articles based on secondary data of these surveys have been published in peer-review journals in epidemiology and public health, especially since the 2000s. For example, around 50 journal articles have used secondary data from one of the two major national household surveys on health in Japan: the Comprehensive Survey of Living Conditions^15^^–^^34^ and the National Nutrition Survey (renamed the National Health and Nutrition Survey in 2003).^17^^–^^19^^,^^25^^,^^32^^,^^35^^–^^63^ Topics covered by the articles using the Comprehensive Survey of Living Conditions include socioeconomic factors,^15^^,^^20^^,^^23^^,^^24^^,^^27^^,^^32^ inequality,^19^^,^^21^^,^^26^^,^^28^ gender differences,^16^^,^^33^ self-rated health,^15^^,^^24^^,^^29^ mental health,^20^^,^^27^^,^^34^ activities of daily living,^33^ and health-related quality of life.^16^^,^^22^ Articles using the National (Health and) Nutrition Survey have examined anemia,^41^^,^^50^ oral health,^45^^–^^47^^,^^49^ dyslipidemia,^52^^,^^55^ hypertension,^37^^,^^60^ diabetes,^62^ weight status and obesity,^35^^,^^40^^,^^42^^–^^44^ and lifestyle-related factors, including tobacco smoking,^45^^–^^47^ dietary intake,^38^^,^^39^^,^^53^^,^^54^^,^^58^^,^^59^^,^^63^ and physical activity.^57^^,^^61^ In some studies, anonymized individual-level records were linked between the two surveys to investigate socioeconomic factors and distribution of health outcomes.^17^^–^^19^^,^^25^^,^^32^

The number of such publications is, however, relatively small for the scale and coverage of the surveys: it falls far behind other nations’ comparable survey data. For example, as of November 10, 2015, systematic searches in PubMed resulted in over 4500 papers published since 1980 using the United States National Health and Nutrition Examination Surveys, while more than 500 papers have been published since 2004 employing the Korea National Health and Nutrition Examination Surveys in South Korea.

## OBSTACLES TO SECONDARY ANALYSIS OF SURVEY DATA

There appear to be two major obstacles to the secondary use of national health survey data in Japan: (i) legal constraints on access to individual-level data and (ii) difficulties in dealing with the disadvantages of secondary data. Regarding the legal constraints, under Item 2, Article 33 of the Statistics Act (Act No. 53, May 23, 2007), the central government strictly controls the use of secondary data of official statistical surveys for scientific purposes. The act stipulates that the head of an administrative body involved in implementing an official statistical survey may provide parties other than administrative organizations with individual-level data of the survey under the following conditions: a high level of public benefit is recognized; the confidentiality of personal information is protected; and public trust is ensured.^64^ Item 2, Article 9 of the Ordinance for Enforcement of the Statistics Act makes a further specification: secondary use of survey data must pertain to the production of statistics and statistical studies for which a public organization calls for public participation, and that organization decides to support all or part of the costs necessary for implementation.^65^ Thus, researchers are able to access individual-level data of national surveys only if their application for providing questionnaire information is related to research projects funded or approved by a public scientific body and is accepted by an administrative organization responsible for a survey. Moreover, in the Guidelines for the Application of Article 33 of the Statistics Act, the Ministry of Internal Affairs and Communications clearly states that use of secondary data of official statistical surveys is restricted to Japan.^66^ This guideline eliminates the possibility of utilization of secondary data by overseas researchers.

Another obstacle to employing existing national health survey data is methodological difficulties in handling the disadvantages of secondary data. Users of secondary data benefit from the convenience and economy of relatively easy access to datasets from nationally representative samples, covering a wide range of topics over a long period.^2^^,^^67^^,^^68^ However, such users are not involved in the process of data collection and therefore have no control over the conditions and quality of data. For example, variables of interest may be lacking from a survey; the measurement methodologies, framing, and wording of survey items may be inconsistent across surveys or change within surveys over time; and causal relationships cannot be directly determined from cross-sectional survey data. Researchers may even prefer using primary or secondary data obtained from large prospective cohort studies in local communities to national survey data—particularly for assessing an association between a health outcome and baseline factors. However, these characteristics of secondary data apply to national health surveys in any country and do not justify their limited use in Japan.

## ASSESSMENT OF RISK FACTORS FOR NON-COMMUNICABLE DISEASES

To illustrate the usefulness of secondary survey data analysis in epidemiology in Japan, I present in this section a few examples from some of my own studies. My colleagues and I applied interdisciplinary approaches in analyzing secondary data of individual records from official statistical surveys in Japan—particularly the National Health and Nutrition Survey and its predecessor, the National Nutrition Survey. Methodological details of the Japanese nutrition surveys are described elsewhere.^69^^,^^70^ Over the past few decades, these surveys have annually measured anthropometric, biochemical, and clinical profiles on individuals. To the best of my knowledge, Japan is the only country that has kept annual electronic records of measured biomarkers on individuals at the national level over such a long period.

The population of Japan underwent a dramatic increase in longevity immediately after World War II. An analysis of published data from vital records showed that the rise in life expectancy at birth during the 1950s and 1960s was largely attributable to decreases in infant mortality from gastroenteritis and pneumonia and in young adult mortality from tuberculosis.^71^ In the late 1960s, the major driver of the increase in longevity shifted to a decrease in adult mortality from non-communicable diseases, such as stroke.^72^ A study based on published statistics of the National Nutrition Surveys for 1956–1980 determined that the decrease in stroke mortality occurred at around the same time as a reduction in average blood pressure at the population level; that finding may partly reflect increased use of antihypertensive drugs in clinical practice under universal health insurance coverage.^73^ Today, life expectancy at birth in Japan is increasing, albeit at a decelerating rate. To further improve population health, consistent and comparable evidence is essential to set priorities in policies and programs for effectively controlling the burden of non-communicable diseases.

### Comparative risk assessment of adult mortality

To investigate the determinants of health and longevity for Japan’s population, my colleagues and I conducted a comparative risk assessment aimed at examining the most important risk factors for death at the national level.^56^^,^^72^ Using an established single comprehensive framework,^74^ we quantified and compared the contributions of 16 preventable risk factors for adult mortality from non-communicable diseases and injuries.

In the first step of the analysis, we collected data related to exposure to risk factors and their causal associations with cause-specific mortality. We estimated exposure to risk factors using secondary data from the dietary questionnaire, the lifestyle questionnaire, and the physical examination of the National Health and Nutrition Survey of 2007. In collaboration with a group of leading epidemiologists in Japan, we collected epidemiological evidence on causal associations from meta-analyses and large-scale prospective studies, such as the Japan Public Health Center-Based Prospective Study.^75^^–^^83^ We calculated population-attributable fractions from these estimates with respect to exposure and causal associations. The population-attributable fractions measured proportional reductions in mortality that would have been achieved if risk factor exposures of a population had shifted to alternative, more favorable counterfactual distributions.

In the second step of the analysis, we multiplied the population-attributable fractions by the number of cause-specific deaths to determine the number of deaths associated with each risk factor. We obtained the number of cause-specific deaths from individual mortality records of vital registration in 2007. To improve the validity, reliability, and comparability of the data on cause-specific mortality, we followed algorithms developed for the Global Burden of Disease Study. The algorithms redistributed ill-defined codes on death certificates, such as those for cardiac arrest, heart failure, and senility, that were not supposed to be the underlying causes of death.^84^

Results of the comparative risk assessment suggested that tobacco smoking and high blood pressure were the two major risk factors for mortality among adults aged 30 years and over in Japan in 2007. Of 834 000 adult deaths from non-communicable diseases and injuries, 129 000 deaths from cancer, cardiovascular diseases, and respiratory diseases were attributable to smoking; 104 000 cardiovascular deaths—largely from stroke and ischemic heart disease among older people—would not have occurred if systolic blood pressure had been maintained at optimal levels. These two risk factors were followed by 52 000 deaths associated with physical inactivity and 34 000 deaths linked to high blood glucose concentration or high dietary sodium intake.

These results are similar to findings in high-income countries, including the United States^85^^,^^86^; however, the contribution of high body mass index (19 000 deaths) was fairly small in Japan. One characteristic of adult mortality in Japan was a relatively large number of deaths from cancer attributable to infectious agents, such as stomach cancer related to *Helicobacter pylori* (31 000 deaths) and liver cancer related to hepatitis C virus infection (23 000 deaths). The mortality burden attributable to infection will, however, decrease in the future because the prevalence of infection from these agents has been declining. The findings of our study have been widely utilized for developing clinical guidelines and health promotion policies at the national and subnational levels.^87^^,^^88^

### Estimation of treatment effects from observational data

As noted above, one study using published statistics found an association between the decrease in mean blood pressure and increased use of blood pressure-lowering drugs in Japan.^73^ However, no analysis had been undertaken of individual records to examine in greater detail the factors behind the decline in population blood pressure. Therefore, by pooling secondary data of the National Nutrition Surveys conducted between 1986 and 2002, my colleagues and I assessed the contribution of antihypertensive medications and lifestyle factors to the decrease in mean systolic blood pressure during that period.^51^

A methodological challenge in that analysis involved estimating the effects of antihypertensive drugs on systolic blood pressure from the observational data of the pooled cross-sectional surveys. It is to be expected that proper medication will lower blood pressure. Nevertheless, simultaneous causality may exist between antihypertensive treatment and blood pressure: at the time of the survey, people may have been receiving antihypertensive medication owing to their high blood pressure, and at the same time the medication would have reduced the blood pressure. Consequently, a simple ordinary least-squares regression of systolic blood pressure on medication use might yield a biased positive association.

Having tried multiple methods, including propensity-score matching, to deal with unobserved treatment selection bias in observational data, we selected an econometric method of the two-stage least-squares regression with an instrumental variable. For an instrumental variable, we adopted the proportion of people—by sex and prefecture of residence—with hypertension who were receiving treatment; we did so under the assumption that this aggregate measure would have no direct relationship with the individuals’ systolic blood pressure. In the first stage of the two-stage least-squares regression, we predicted the probability of undergoing treatment from the logistic regression of receiving antihypertensive medication on the instrumental variable and covariates (such as body mass index, cigarette smoking, alcohol consumption, regular exercise, and daily salt intake). In the second stage of the model, we conducted an ordinary least-squares regression to determine the associations of systolic blood pressure with the predicted probability of being treated and all of the explanatory variables used in the first stage except the instrumental variable. We used estimated regression coefficients and their variances to decompose the decrease in mean systolic blood pressure between 1986 and 2002 into contributions of the explanatory variables.

The results of our study suggested that the decline in mean systolic blood pressure from the late 1980s to the early 2000s may be partly explained by the increased use of antihypertensive drugs—particularly among older patients with hypertension—and to a lesser extent by reduced dietary salt intake. However, a substantial part of the blood pressure reduction was unexplained. That may have been partly due to the use of the single measurement of blood pressure in the analysis and the presence of unobserved explanatory variables in the survey, such as socioeconomic status and individual nutrient intake.

### Comparable evidence on management of high blood pressure

To assess the performance of health systems in controlling risk factors, it is necessary to obtain comparable evidence. A number of studies have examined the awareness, treatment, and control of hypertension in the general population using secondary data from national health surveys.^89^^–^^104^ However, their results are not always directly comparable, largely because of inconsistencies in measurement methods and analytic strategies. For example, the number of blood pressure readings varies across surveys over time: the Japan National Nutrition Survey took only a single blood pressure measurement until it started measuring twice in 2000; the United States National Health and Examination Survey measured blood pressure six times in 1988–1994 and three times in 1999–2014. Currently, blood pressure is measured at least twice from a participant in many national health and examination surveys because the first measurement tends to be higher than usual.^60^^,^^91^^,^^96^ It appears to be normal practice in studies to discard the unstable first reading and use the average of subsequent readings when more than one measurement is taken.^10^^,^^91^^,^^103^ Nevertheless, some government publications have employed the average of all available blood pressure measurements, including the first one.^105^^,^^106^

Such discordances in measurements and analytic protocols across studies may confuse and mislead readers about the performance of health systems in the control of population blood pressure. Therefore, to achieve comparable evidence for different countries over time, my colleagues and I estimated indicators for the management of hypertension under consistent definitions from the secondary data of national health examination surveys.^60^

The key part of our study was exploration of access to individual-level data. We used several electronic databases to search systematically for information on national health examination surveys from various parts of the world. We reviewed journal articles and survey reports to determine whether the surveys satisfied the criteria for inclusion in our study: (i) a random sample of adults of a whole country; (ii) inclusion of both sexes; and (iii) data on blood pressure measurements, diagnosis of hypertension, and use of antihypertensive medications. The surveys in 73 of 193 World Health Organization member states satisfied these criteria at the time of our study. After downloading publicly available datasets or officially requesting data from the institutions that conducted the surveys, we finally obtained anonymized individual-level data from 20 countries: seven low-income, nine middle-income, and four high-income countries. Data for multiple years were available for Japan, the United Kingdom, and the United States.

Using consistent definitions, we estimated for each country the prevalence of hypertension—defined as systolic blood pressure ≥140 mm Hg or currently receiving antihypertensive medication—and the proportion of hypertensive individuals whose condition was diagnosed, treated, or controlled with medication. Our results showed that the prevalence of hypertension was substantial in some low- and middle-income countries, and blood pressure control in hypertensive individuals was particularly poor in Albania, Armenia, Iran, and Turkey. We also found that the treatment and control coverage of high blood pressure was substantially lower in Japan than in the United States; progress in managing hypertension with medication over time was slower in Japan than in the United Kingdom and United States.

## CONCLUSIONS AND FUTURE DIRECTIONS

In this paper, I have discussed the challenges and achievements of secondary data analysis of national health surveys in Japan. I conclude with two recommendations about future directions for advancing health metrics and evaluation research in this country.

First, to increase the usefulness of health survey data, panels of experts and departments of the ministry responsible for survey implementation need to make greater efforts to revise survey designs and questionnaires so that they are comparable in quality with those of other countries. For example, instead of the National Health and Nutrition Survey being implemented annually, I believe it would be better to conduct it with an expanded sample every 3 years in conjunction with the large-scale survey of the Comprehensive Survey of Living Conditions. That way, researchers would be able to perform a more statistically powerful analysis on secondary data of the National Health and Nutrition Survey; they would also be able to extend their analysis by using data linked with the health questionnaire of the Comprehensive Survey of Living Conditions. Although this redesign of the survey would require further reinforced coordination among the relevant authorities and impose an additional burden on them, in the long term, it would benefit survey administrators if that revision reduced implementation costs and the workload on survey interviewers while concurrently increasing the response rates. Substantial improvements in quality could be expected through allowing sufficient time and resources for planning, implementation, and assessment between the surveys.

Second, interdisciplinary research in population health should be more strongly encouraged in departments of public health and epidemiology at universities and research institutes in Japan. Researchers need to collaborate and interact across academic disciplines as teams to exchange ideas and advanced analytic techniques for their front-line research with secondary data. A world-class research environment for population health sciences should be established in Japan to maximize the interests and knowledge of leading scientists and also to provide students with training opportunities for understanding the complex system of health and wellbeing of populations. Such institutional reforms will ultimately lead to advances in knowledge and techniques for secondary survey data analysis.

National health surveys in Japan are an invaluable asset, and findings from secondary analyses of these surveys could provide important suggestions to improve the health of people around the world. I hope this paper encourages many epidemiologists in Japan to undertake analyses of secondary survey data and disseminate high-quality evidence from this country to the global community of population health researchers and policymakers.
